# Retrospective analysis of cohort database: Phenotypic variability in a large dataset of patients confirmed to have homozygous familial hypercholesterolemia

**DOI:** 10.1016/j.dib.2016.04.004

**Published:** 2016-04-11

**Authors:** Frederick J. Raal, Barbara Sjouke, G. Kees Hovingh, Barton F. Isaac

**Affiliations:** a Department of Medicine, Faculty of Health Sciences, University of the Witwatersrand, Johannesburg, South Africa; b Department of Vascular Medicine, Academic Medical Center, University of Amsterdam, PO Box 22660, 1100 DD Amsterdam, Netherlands; c Department of Vascular Medicine, Academic Medical Center, University of Amsterdam, Meibergdreef 9, 1100 DD Amsterdam, Netherlands; d Medical Affairs, Sanofi Genzyme, 500 Kendall Street, Cambridge, MA 02142, USA

**Keywords:** Homozygous familial hypercholesterolemia, Cholesterol, Phenotype

## Abstract

These data describe the phenotypic variability in a large cohort of patients confirmed to have homozygous familial hypercholesterolemia. Herein, we describe the observed relationship of treated low-density lipoprotein cholesterol with age. We also overlay the low-density lipoprotein receptor gene (*LDLR*) functional status with these phenotypic data. A full description of these data is available in our recent study published in *Atherosclerosis*, “Phenotype Diversity Among Patients With Homozygous Familial Hypercholesterolemia: A Cohort Study” (Raal et al., 2016) [1].

**Specifications Table**

**Table:** 

Subject area	*Epidemiology*
More specific subject area	*Lipidology*
Type of data	*Figures, table*
How data was acquired	*Retrospective analysis of three homozygous familial hypercholesterolemia cohort databases*
Data format	*Analyzed*
Experimental factors	*Age, LDL-C, LDLR functional status*
Experimental features	*Age of patients plotted vs treated LDL-C. LDLR functional status added to secondary plot*
Data source location	*The Netherlands, South Africa*
Data accessibility	*Data is with this article.*

**Value of the data**

• The stratification of patients according to LDL receptor (*LDLR*) functionality adds further insight to these data.
• Application of this type of assessment to other homozygous familial hypercholesterolemia (HoFH) cohorts could add insight to phenotype and genotype variability.
• These data describe the relationship of age and treated low-density lipoprotein cholesterol (LDL-C).

## 1 Data

Data are derived from 3 recent international studies comprising a total of 167 HoFH patients [1].

**Fig. 1:**
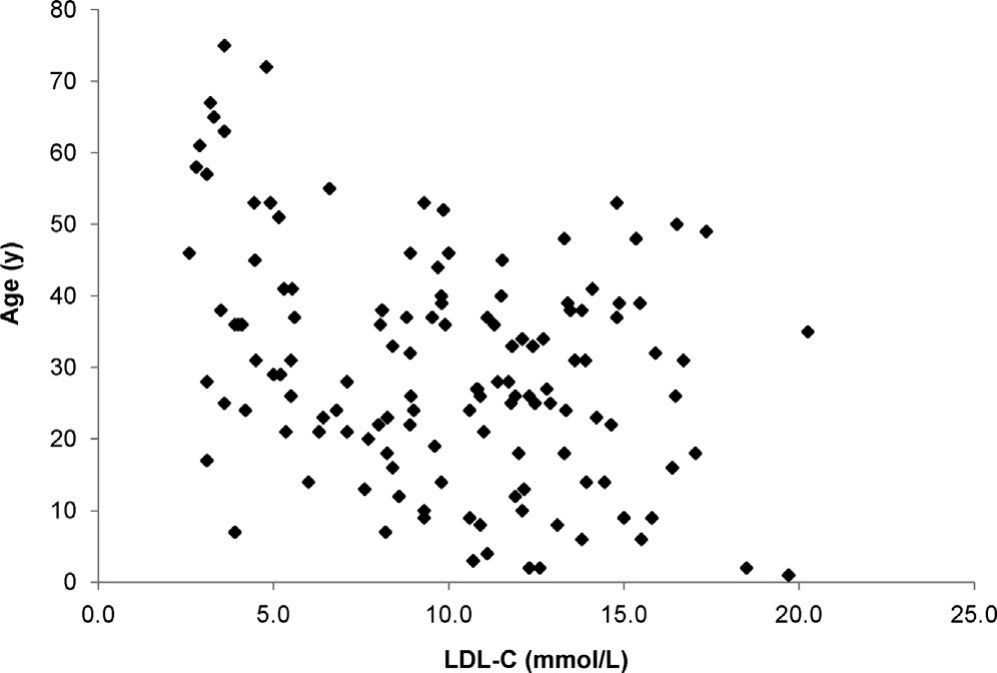
Patient age vs tLDL-C values among patients in all cohorts with available tLDL-C data (*n*=134)**.** LDL-C indicates low-density lipoprotein cholesterol; tLDL-C, treated low-density lipoprotein cholesterol.

**Fig. 2:**
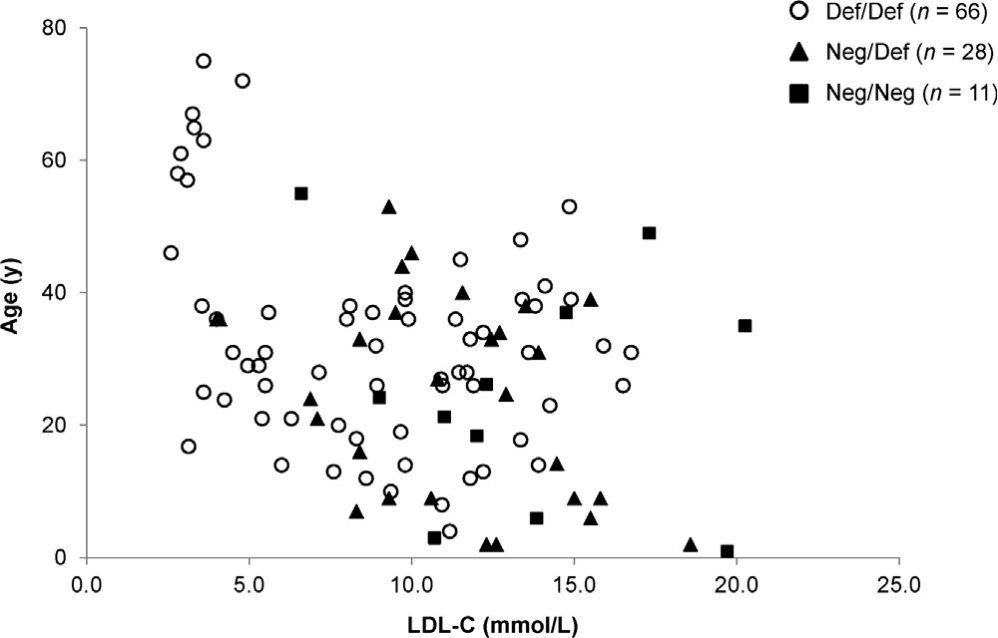
Patient age vs tLDL-C values among patients in all cohorts stratified by *LDLR* functional status**.** Def/Def indicates defective/defective; *LDLR*, low-density lipoprotein receptor gene; Neg/Def, negative/defective; Neg/Neg, negative/negative; tLDL-C, treated low-density lipoprotein cholesterol.

The relationship between patient age and treated LDL-C (tLDL-C) values is presented in the form of scatter plot diagrams (Fig. 1, Fig. 2).

**Fig. 3:**
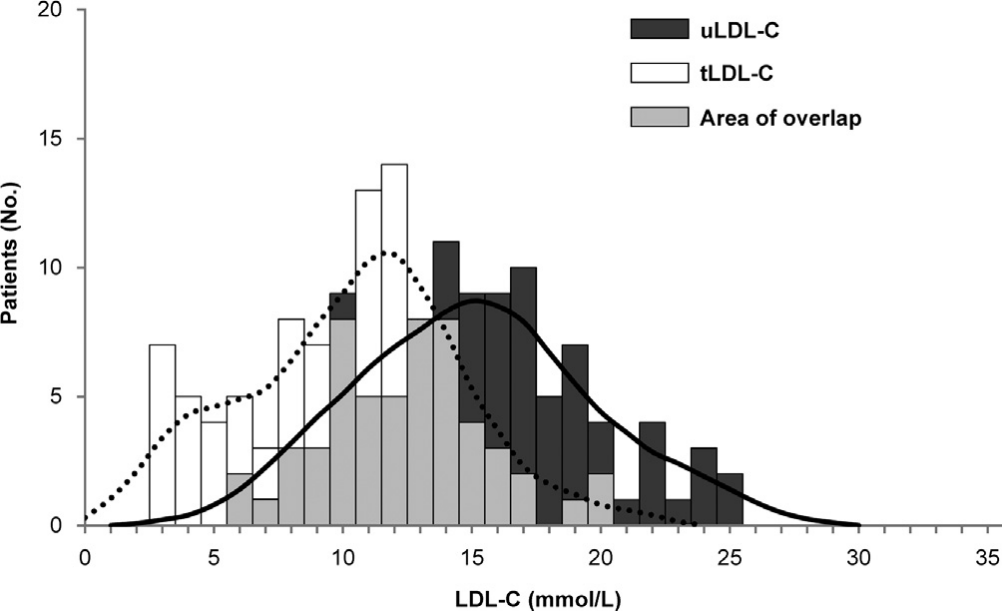
Distribution of LDL-C values by treatment status for patients in all cohorts with both uLDL-C and tLDL-C data (*n*=102)**.** LDL-C indicates low-density lipoprotein cholesterol; tLDL-C, treated low-density lipoprotein cholesterol; uLDL-C, untreated low-density lipoprotein cholesterol. Dotted line: distribution of patients with a particular tLDL-C; solid line: distribution of patients with a particular uLDL-C.

Distribution curves of the LDL-C values for the 102 patients who had paired untreated LDL-C (uLDL-C) and tLDL-C are shown in Fig. 3.

Table 1 lists the criteria endorsed by the European Atherosclerosis Society (EAS) Consensus Panel on Familial Hypercholesterolemia [2].

## 2 Experimental design, materials and methods

The first of the three datasets used for the present analysis consists of the baseline data pertaining to patients enrolled in a phase 3, multicentre, international, randomised, double-blind, placebo-controlled trial (the Genzyme [GZ] HoFH study, ClinicalTrials.gov number NCT00607373, sponsored by Sanofi Genzyme) comparing treatment with mipomersen vs placebo in patients with HoFH [3]. It should be noted that the baseline LDL-C levels derived from this study reflect LDL-C levels prior to treatment with mipomersen. The second dataset is derived from a published retrospective chart review of patients treated at two specialised lipid clinics in South Africa (SA study) between 1972 and 2009 [4]. The third dataset is the published analysis of data derived from the national database of patients with HoFH in the Netherlands, compiled by the Academic Medical Center in Amsterdam (AMC study), a nationwide DNA diagnostic center where patients in the Netherlands are referred for molecular diagnosis of familial hypercholesterolemia [5].

### 2.1 Diagnostic criteria

Diagnostic criteria for HoFH used in the GZ HoFH study and SA study were identical, and largely mirror those of the EAS Consensus Panel: genetic confirmation of two mutant alleles at the *LDLR* gene locus or clinical diagnosis based on untreated LDL-C levels >13 mmol/L (500 mg/dL) in addition to either xanthoma(s) observed before 10 years of age or evidence of heterozygous FH in both parents [3], [4]. Diagnostic criteria for the AMC study involved confirmation of pathogenic mutations for autosomal-dominant FH, specific to monogenic manifestations [5].

### 2.2 Exclusion criteria

Subjects meeting the following criteria were excluded: (1) subjects actively undergoing lipoprotein apheresis, (2) subjects with genetic confirmation of a form of HoFH that did not directly involve the *LDLR* gene (e.g., *APOB*), and (3) subjects who were deceased.

### 2.3 Phenotypic assessment

LDL-C levels were obtained at each center from medical records. Treated LDL-C (tLDL-C) refers to the LDL-C level while the patient was taking the maximally tolerated available lipid-lowering therapy (LLT).

Patient age in the GZ HoFH study cohort was derived from the case report forms at baseline enrollment. Patient age in the AMC cohort was published online as supplemental data to the original publication [5]. To be consistent with the GZ HoFH cohort, a conservative age was used for patients in the SA lipid clinics, based on the year that enrollment began in the GZ HoFH study (i.e., 2007), instead of using patient age at the time of this analysis.

### 2.4 Molecular assessment

Molecular assessment was undertaken based on classification of *LDLR* mutations into one of six categories: (1) defective/defective, (2) defective/negative, (3) negative/negative, (4) defective/unclassified, (5) negative/unclassified, or (6) unclassified/unclassified. An LDL receptor mutation designated as “negative” is associated with <2% of LDL uptake in cultured fibroblasts; a receptor mutation designated as “defective” is associated with 2–25% of normal uptake [6]. If the receptor status was not reported or was unknown in the study publication, it was considered to be unclassified.

### 2.5 Statistical analysis

The LDL-C distribution curves demonstrate the overlap of uLDL-C and tLDL-C values. For the comparison of the AMC cohort and combined SA and GZ HoFH baseline cohorts, SAS software was employed to perform a two-sample independent *t*-test. This comparison was chosen because patients in the AMC study had a molecular diagnosis, whereas the SA and the GZ HoFH baseline cohorts had predominantly a phenotypic diagnosis with confirmation of HoFH using molecular diagnosis, if available. Comparisons of the AMC study with the SA+GZ HoFH studies were conducted for both uLDL-C and tLDL-C values. The relationship between patient age and tLDL-C values is presented in the form of scatter plot diagrams (Fig. 1, Fig. 2). Data regarding patient age at the time of the recording of uLDL-C values were not always available.

## Figures and Tables

**Table 1:** Clinical and genetic criteria for the diagnosis of homozygous familial hypercholesterolemia.

**Clinical criteria**	**Genetic criteria**
Untreated LDL-C level >13 mmol/L (500 mg/dL)	Genetic confirmation of 2 mutant alleles at the *LDLR, APOB, PCSK9,* or *LDLRAP1* gene locus
OR
Treated LDL-C level ≥8 mmol/L (300 mg/dL)^a^
Accompanied by either: a. Cutaneous or tendinous xanthoma before age 10 years, or b. Untreated, elevated LDL-C levels consistent with HeFH in both parents

The table is adapted with permission from Cuchel et al. [2].

HeFH, heterozygous familial hypercholesterolemia; HoFH, homozygous familial hypercholesterolemia; LDL-C, low-density lipoprotein cholesterol; LDLR, low-density lipoprotein receptor gene; APOB, familial defective apolipoprotein B gene; PCSK9, proprotein convertase subtilisin/kexin type 9 gene; LDLRAP1, LDL receptor adaptor protein 1 gene.

a LDL-C levels reflect a standard range; lower levels of LDL-C may be seen in HoFH, particularly in children and treated patients.
